# Wound Healing in Mice with High-Fat Diet- or *ob* Gene-Induced Diabetes-Obesity Syndromes: A Comparative Study

**DOI:** 10.1155/2010/476969

**Published:** 2011-01-20

**Authors:** Oliver Seitz, Christoph Schürmann, Nadine Hermes, Elke Müller, Josef Pfeilschifter, Stefan Frank, Itamar Goren

**Affiliations:** Pharmazentrum Frankfurt/ZAFES, Institut für Allgemeine Pharmakologie und Toxikologie, Klinikum der Johann Wolfgang Goethe-Universität, Theodor-Stern-Kai 7, 60590 Frankfurt am Main, Germany

## Abstract

In the past, the genetically diabetic-obese *diabetes/diabetes* (*db/db*) and *obese/obese* (*ob/ob*) mouse strains were used to investigate mechanisms of diabetes-impaired wound healing. Here we determined patterns of skin repair in genetically normal C57Bl/6J mice that were fed using a high fat diet (HFD) to induce a diabetes-obesity syndrome. Wound closure was markedly delayed in HFD-fed mice compared to mice which had received a standard chow diet (CD). Impaired wound tissue of HFD mice showed a marked prolongation of wound inflammation. Expression of vascular endothelial growth factor (VEGF) was delayed and associated with the disturbed formation of wound margin epithelia and an impaired angiogenesis in the reduced granulation tissue. Normal wound contraction was retarded and disordered. Wound disorders in obese C57Bl/6J mice were paralleled by a prominent degradation of the inhibitor of NF*κ*B (I*κ*B-*α*) in the absence of an Akt activation. By contrast to impaired wound conditions in *ob/ob* mice, late wounds of HFD mice did not develop a chronic inflammatory state and were epithelialized after 11 days of repair. Thus, only genetically obese and diabetic *ob/ob* mice finally developed chronic wounds and therefore represent a better suited experimental model to investigate diabetes-induced wound healing disorders.

## 1. Introduction

Diabetes mellitus has seen an extensive global increase, which had been predicted nearly a decade ago [1]. Diabetic skin ulcerations represent a severe complication of the disease, and more important, a still unmet medical problem associated with significant mortality [2, 3]. The lifetime risk for any diabetic patient to develop this complication is about 15% [4]. Moreover, diabetic ulcers have a poor prognosis and the 3-year survival rate after amputations are only between 50 and 59% [5, 6]. Predisposing factors (neuropathy, ischemia), which lead to foot ulceration [7], are all but impossible to imitate using the respective animal models of wound healing. Nevertheless, diabetic and obese rodents have long been used as animal models to unravel molecular and cellular mechanisms that might form the basis of or at least contribute to diabetes-disturbed wound conditions.

In particular, the genetically obese (*ob/ob*) and diabetes (*db/db*) mouse strains [8] had been favored as animal models of diabetes-impaired skin repair [9–13]. Upon wounding, *ob/ob* and *db/db* mice develop severely disordered wound conditions. Seminal studies using these animals identified substantial mechanisms that contributed to the observed failure of tissue regeneration. The most severe defects in terms of impaired reepithelialization and granulation tissue formation were strongly associated with a loss of function of diverse growth factors that drive keratinocyte, fibroblast and endothelial cell functions [10–12, 14–16] in the presence of a greatly augmented wound inflammatory response [13, 17, 18].

It is noteworthy to allude to the fact that these earlier studies in *ob/ob* and *db/db* mice were focused mainly on the diabetic phenotype of the animals. However, it also turned out that the diabetic phenotype of these animals appears to be functionally connected to their huge adipose tissue mass [19]. At present, a novel concept has evolved that convincingly implicates an adipose tissue-driven activation of macrophages as the central cause of insulin-resistance under conditions of severe obesity [20–22]. This basic concept appeared to also prove true for diabetes-impaired healing in obese mice, as specific depletion of macrophages from sites of injury markedly improved tissue repair upon wounding and transplantation [18, 23]. 

By contrast to the intensive use of genetically diabetic and obese mice in terms of wound repair, there are no data available to date that are based on a diet-induced diabetic and obese phenotype in wild-type mice. Here, we wounded genetically normal C57Bl/6J mice which had been rendered obese and diabetic using a high-fat diet (HFD) for 27 weeks. Consistent with findings in *ob/ob* mice, HFD-fed animals exhibited a delayed wound closure that was associated with impaired wound reepithelialization and contraction, a prolonged wound inflammation and a markedly disturbed activation of central intracellular signaling pathways. Our data suggest that the genetically diabetic *ob/ob* mouse represents a suitable animal model to describe mechanisms of impaired wound healing under conditions of an obesity-associated insulin-resistance.

## 2. Materials and Methods

### 2.1. Animals

Female C57Bl/6J (wild-type) and C57Bl/6J-*ob/ob* mice were obtained from The Jackson Laboratories (Bar Harbor, ME) and maintained under a 12 h light/12 h dark cycle at 22°C.

### 2.2. Feeding of Mice

At the age of 6 weeks (for C57Bl/6J) or 12 weeks (for C57Bl/6J-*ob/ob*), mice were caged individually and monitored for body weight. For the high-calorie diet study, six-week-old C57Bl/6J wild-type mice (*n* = 30) were maintained *ad libitum* on water and a high-fat diet (HFD) (D12331, Research Diets Inc., New Brunswick, NJ) for 190 days to induce an obese and diabetic phenotype in the animals. The HFD contained high-fat (35.8%), protein (23.0%) and carbohydrate (35.5%). The calories provided by fat, protein and carbohydrate were 58.0%, 16.4% and 25.5%, respectively. C57Bl/6J control mice received water and a control chow diet (CD) *ad libitum* for 190 days with calories provided by fat (11%), protein (23%) and carbohydrate (65%). C57Bl/6J-*ob/ob *mice were fed with a CD throughout the experiment.

### 2.3. Oral Glucose Tolerance Test (OGT)

CD- and HFD-fed mice or *ob/ob* mice were starved for 16 h. Mice were subsequently applied orally with glucose (1.5 g per kg body weight) by gastrogavage. Blood glucose levels were determined before and 60 min following glucose application.

### 2.4. Wounding of Mice

Following the feeding period of 190 days, CD- and HFD-fed mice were 33 weeks old at wounding. C57BL/6J-*ob/ob *mice were wounded at the age of 12 weeks. Wounding of mice was performed as described previously [24, 25]. Briefly, mice were anaesthetized with a single i.p. injection of ketamine (80 mg/kg body weight)/xylazine (10 mg/kg body weight). The hair on the back of each mouse was cut, and the back was subsequently wiped with 70% ethanol. Six full-thickness wounds (5 mm in diameter, 3-4 mm apart) were made on the back of each mouse by excising the skin and the underlying *panniculus carnosus*. The wounds were allowed to form a scab. Skin biopsy specimens were obtained from the animals 1, 3, 5, 7, and 11 days after injury. At each time point, an area which included the scab, the complete epithelial and dermal compartments of the wound margins, the granulation tissue, and parts of the adjacent muscle and subcutaneous fat tissue was excised from each individual wound. As a control, a similar amount of skin was taken from the backs of nonwounded mice. For each experimental time point, tissue from four wounds each from four animals (*n* = 16 wounds, RNA analysis) and from two wounds each from four animals (*n* = 8 wounds, protein analysis) were combined and used for RNA and protein preparation. Nonwounded back skin from four animals served as a control. All animal experiments were carried out according to the guidelines and were approved by the local Animal Ethics Review Board.

### 2.5. Analysis of Serum from CD- or HFD-Fed and *ob/ob* Mice

Blood glucose levels were determined using the AccuTrend sensor (Roche Biochemicals, Mannheim, Germany). Serum insulin, leptin and tumor necrosis factor (TNF-*α*) were analyzed by Luminex (Biotrend, Düsseldorf, Germany) as described by the manufacturer.

### 2.6. RNA Isolation and RNase Protection Analysis

RNA isolation and RNase protection assays were carried out as described previously [25, 26]. If not indicated otherwise, every experimental time point depicts 12 wounds (*n* = 12) isolated from four individual mice (*n* = 4) for all RNase protection assays analyzing wound tissue samples. All samples were quantified using PhosphoImager PSL counts per 15 *μ*g of total wound RNA. Glyceraldehyde phosphate dehydrogenase (GAPDH) hybridization was used as a loading control. The cDNA probes were cloned using RT-PCR. The probes corresponded to nt 425 (exon 1) to 170 (exon 2) (for lysozyme M, GenBank accession number M21047), nt 816 to 1481 (for lipocalin, X81627), nt 912 to 1183 (for *α*-smooth muscle actin (*α*-SMA), BC064800.1) or nt 163–317 (for GAPDH, NM002046) of the published sequences.

### 2.7. Immunohistochemistry

Mice were wounded as described above. Animals were sacrificed at day 5 and 7 after injury. Biopsies from skin wounds were isolated from the back, fixed in formalin or in zinc fixative solution (0.05% CaAc·2H_2_O, 0.5 ZnAc·2H_2_O, 0.5% ZnCl_2_ in 0.1 M Tris-HCl, pH 7.4) and subsequently embedded in paraffin. Sections (4 *μ*m) were subsequently incubated over night at 4°C with antisera raised against murine F4/80 (AbD Serotec, Düsseldorf, Germany), Ly-6G (Gr-1) (BD Pharmingen, Heidelberg, Germany), VEGF (Santa Cruz, Heidelberg, Germany), CD31 (Chemicon, Eschborn, Germany), or *α*-SMA (Sigma, Taufkirchen, Germany). Primary antibodies were detected using a biotinylated secondary antibody. The slides were subsequently stained with the avidin-biotin-peroxidase complex system (Santa Cruz) using 3,3-diaminobenzidine-tetra-hydrochloride or Fast Red Substrate-Chromogen System (Dako, Hamburg, Germany) as chromogenic substrates. Finally, sections were counterstained with hematoxylin and mounted.

### 2.8. Preparation of Protein Lysates and Western Blot Analysis

Skin and wound tissue biopies were homogenized in lysis buffer (1% Triton X-100, 137 mM NaCl, 10% glycerol, 1 mM dithiothreitol, 10 mM NaF, 2 mM Na_3_VaO_4_, 5 mM ethylenediaminetetraacetic acid, 1 mM phenylmethylsulfonylfluoride, 5 ng/ml aprotinin, 5 ng/ml leupeptin, 20 mM Tris/HCl, pH 8.0) and cleared by centrifugation. Protein concentrations were determined using the BCA Protein Assay Kit (Pierce Inc., Rockford, IL, USA). Fifty micrograms of total protein from skin lysates were separated using SDS gel electrophoresis. After transfer to a nitrocellulose membrane, specific proteins were detected using antisera raised against F4/80 (AbD Serotec), Ly-6G (Gr-1) (BD Pharmingen), cyclooxygenase (Cox)-2 (Cayman, Ann Arbor, MI, USA), *α*-SMA (Dako, Glostrup, Denmark), phospho-signal transducer and activator of transcription (STAT)-3 (Y705), phospho-Akt (S473) (Cell Signalling, Frankfurt, Germany), inhibitor of nuclear factor *κ*B (I*κ*B)-*α* (Santa Cruz, Heidelberg, Germany) and *β*-actin (Sigma). A secondary antibody coupled to horseradish peroxidase and the enhanced chemiluminescence (ECL) detection system was used to visualize the proteins. Phenylmethylsulfonyl fluoride, dithiothreitol, aprotinin, NaF and Na_3_VaO_4_ were from Sigma. Leupeptin and ocadaic acid were from BioTrend (Köln, Germany). The ECL detection system was obtained from Amersham (Freiburg, Germany).

### 2.9. Enzyme-Linked Immunosorbent Assay (ELISA)

Quantification of murine IL-1*β*, macrophage inflammatory protein (MIP)-2, or vascular endothelial growth factor (VEGF)_165_ protein was performed using the respective murine Quantikine ELISA kits (R&D Systems, Wiesbaden, Germany) according to the instructions of the manufacturer.

### 2.10. Analysis of Neoepithelia

Areas of neoepithelia were determined using the ImageJ analysis programme (http://rsb.info.nih.gov/ij/).

### 2.11. Statistical Analysis

Data are shown as means ± SD. Data analysis was carried out using the unpaired Student's *t* test with raw data. Statistical comparison between more than two groups was carried out by analysis of variance (ANOVA, Dunnett's method).

## 3. Results

### 3.1. High-Fat Diet (HFD) Induces Obesity and Insulin Resistance and Delays Normal Wound Closure

**Figure 1:**
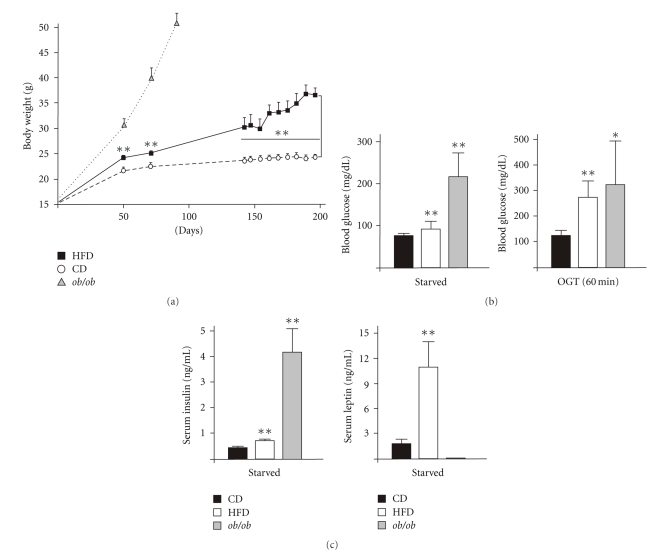
Body weight and diet-induced metabolic changes in mice. (a) Weight gain in CD-fed, HFD-fed or *ob/ob* mice as indicated. (b) CD-fed, HFD-fed and *ob/ob* mice were starved for 16 h. Basal blood glucose levels (left panel) and blood glucose levels (60 min) during an OGT (right panel) were determined. (c) CD-fed, HFD-fed and *ob/ob* mice were starved for 16 h. Serum insulin (left panel) and leptin (right panel) were determined. **P < .01; *P < .05 versus CD-fed mice. Bars indicate the mean ± SD obtained from 26 individual animals.

C57BL/6J wild-type mice were fed using a HFD, which provides 58% of calories as saturated fatty acids for a period of 190 days. Mice were 6 weeks old, when feeding was started. Animals of the control group received a standard control chow diet (CD). The long-term uptake of the HFD caused a significant increase in body weight (Figure 1(a)). The HFD-induced obesity was paralleled by the development of a distinct insulin-resistance in the mice. Starved mice of the HFD group exhibited elevated levels of basal blood glucose (Figure 1(b), left panel) and a severely impaired clearance of acutely increased blood glucose levels during an OGT (Figure 1(b), right panel). The observed impaired glucose tolerance in HFD-treated mice was accompanied by a hyperinsulinaemic state and markedly raised levels of circulating leptin (Figure 1(c)), which both represent well-described features of a developing HFD-induced type 2 Diabetes mellitus in mice [27]. Inline with published data, *ob/ob* mice were severely insulin resistant (Figure 1(b)) and hyperinsulinaemic [28] (Figure 1(c), left panel). Due to the functional loss of the *ob* gene product leptin [29], *ob/ob* mice were deficient of circulating leptin (Figure 1(c), right panel). 

**Figure 2:**
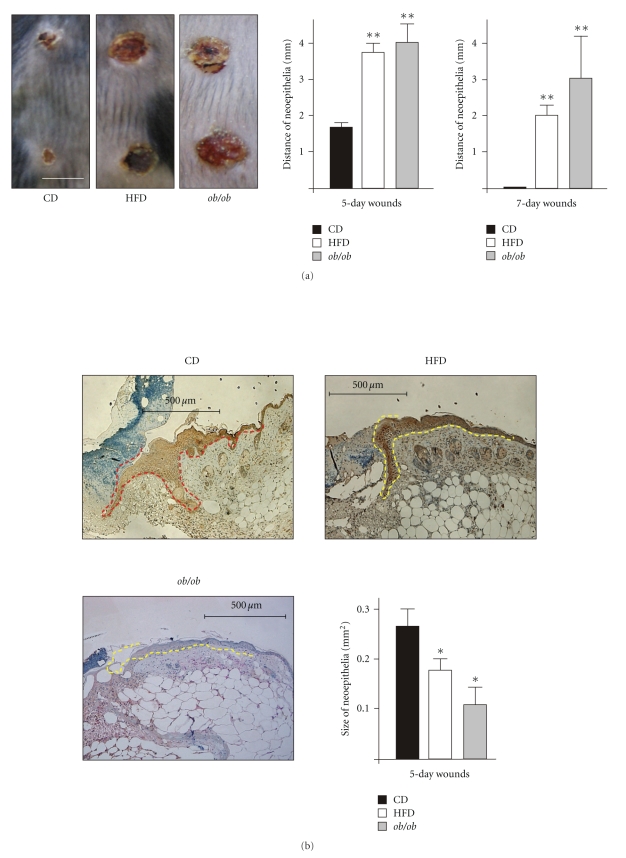

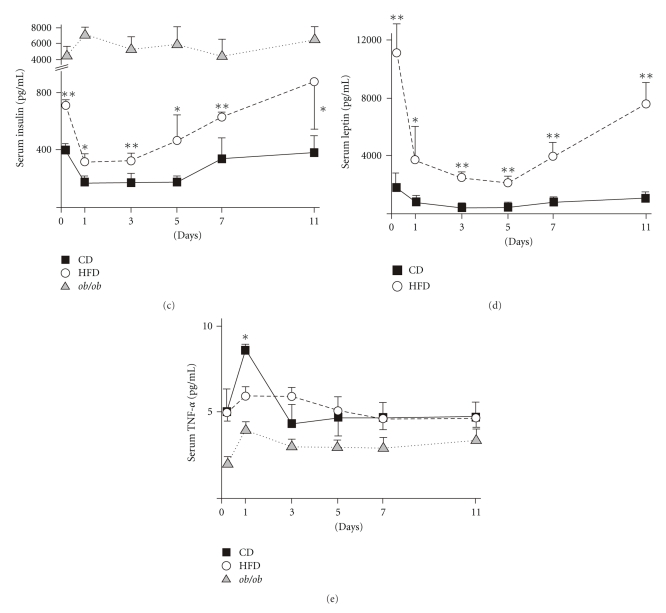
Wound closure and systemic effects of wounding. (a) 5-day wounds in CD-fed, HFD-fed and *ob/ob* mice (left panel). Distances of neoepithelia in cross-sectional areas of 5-day and 7-day wounds of CD-fed, HFD-fed and *ob/ob* mice (right panels). (b) Histologies showing wound neoepithelia of CD, HFD or *ob/ob* mice as indicated. Scale bars = 500 *μ*m. The epithelial margins are indicated by a *yellow line*. *Panel*: areas of wound margin neoepithelia in CD, HFD and *ob/ob* mice. **P < .01 versus CD-fed mice. Bars indicate the mean ± SD obtained from 8 wounds (*n* = 8) from 4 individual animals (*n* = 4). (c) Serum insulin, (d) Leptin and (e) TNF-*α* levels in CD-fed, HFD-fed or *ob/ob* mice before wounding and during different days of healing as indicated. **P < .01; *P < .05 versus CD-fed mice. Each single experimental time point depicts the mean ± SD obtained from 4 individual animals (*n* = 4).

**Figure 3:**
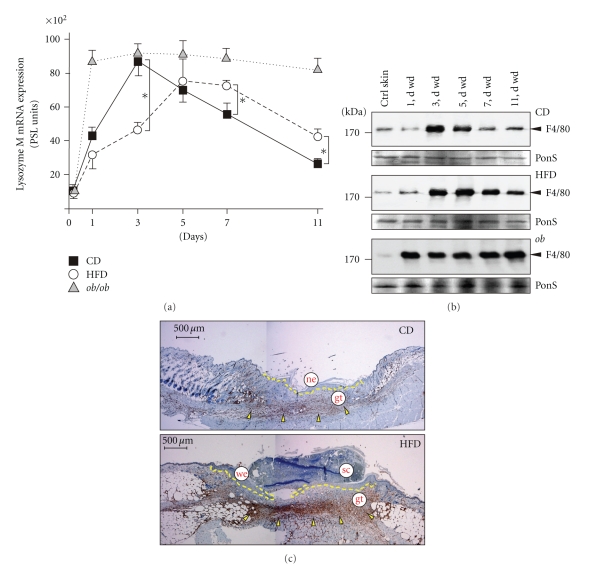
Macrophages in wound tissue. (a) Expression of lysozyme M mRNA in nonwounded back skin and wound tissue isolated from CD-fed, HFD-fed or *ob/ob* mice. The time after injury is indicated. *P < .05 as indicated by the brackets. Each single experimental time point depicts the mean ± SD obtained from 12 wounds (*n* = 12) isolated from 4 individual animals (*n* = 4). (b) Immunoblots showing the presence of F4/80 protein in nonwounded (ctrl) and wounded (1 to 11 d wd) skin in CD, HFD and *ob/ob* mice as indicated. Each time point depicts 8 wounds from (*n* = 8) from 4 individual mice (*n* = 4). A Ponceau S (PonS) staining of the immunoblots is shown to control equal loading. (c) Sections from 7-day wound tissue isolated from CD and HFD mice were stained for macrophage-specific F4/80 protein (*brown colour*) as indicated. F4/80-positive signals were highlighted by *arrows*. The epithelial margins are indicated by a *yellow line*. Scale bar = 500 *μ*m. gt, granulation tissue; ne, neoepithelium; sc, scab; we, wound margin epithelia.

**Figure 4:**
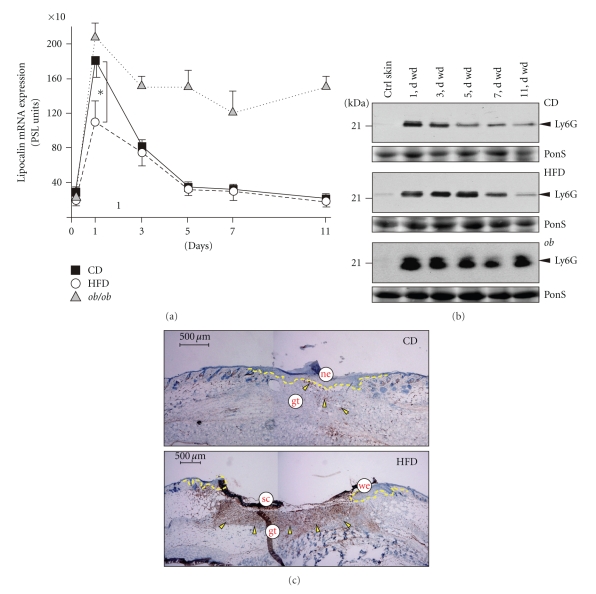
Neutrophils in wound tissue. (a) Expression of lipocalin mRNA in nonwounded back skin and wound tissue isolated from CD-fed, HFD-fed or *ob/ob* mice. The time after injury is indicated. *P < .05 as indicated by the bracket. Each single experimental time point depicts the mean ± SD obtained from 12 wounds (*n* = 12) isolated from 4 individual animals (*n* = 4). (b) Immunoblots showing the presence of Ly6G protein in nonwounded (ctrl) and wounded (1 to 11 d wd) skin in CD, HFD and *ob/ob* mice as indicated. Each time point depicts 8 wounds from (*n* = 8) from 4 individual mice (*n* = 4). A Ponceau S (PonS) staining of the immunoblots is shown to control equal loading. (c) Sections from 7-day wound tissue isolated from CD and HFD mice were stained for neutrophil-specific Ly6G protein (*brown colour*) as indicated. Ly6G-positive signals were indicated by *arrows*. The epithelial margins are indicated by a *yellow line*. Scale bar = 500 *μ*m. gt, granulation tissue; ne, neoepithelium; sc, scab; we, wound margin epithelia.

We started wounding of mice, when we had assured the presence of a combined overweight/obese and diabetic phenotype in the HFD-feeding group (Figure 1). Wounding was carried out in HFD-induced obese and diabetic mice, CD-fed lean mice and in genetically obese and diabetic *ob/ob* mice. After wounding, we generally observed a marked delay in acute wound closure in mice with an obese and diabetic phenotype (HFD, *ob/ob*). Five days upon injury, impaired wounds in HFD-fed and *ob/ob* mice were increased in diameter (Figure 2(a), left panel). This finding was supported by histologic analyses of wound cross-sectional areas, which demonstrated an enlarged distance of wound margin neoepithelia (Figure 2(a), right panel). In support of these data, wound margin epithelia of HFD and *ob/ob* mice were also reduced in size (Figure 2(b)). At day 7 after wounding, wounds of CD mice were completely reepithelialized, whereas epithelialization was still incomplete in HFD mice (Figure 2(a), right panel and Figures 3(c) and 4(c) HFD). In addition, wounds of *ob/ob* mice were impaired at day 5 and 7 after wounding (Figure 2(a), middle and right panel) and even after 11 days of healing (data not shown). At this time point, wounds of CD and HFD mice were completely closed (data not shown).

### 3.2. Skin Injury Changes Levels of Circulating Mediators

Remarkably, wounding of skin tissue also mediated marked systemic effects. The levels of circulating insulin showed a distinct reduction upon wounding in CD- and HFD-treated mice, but not *ob/ob* mice (Figure 2(c)). Furthermore, circulating leptin was reduced in CD and HFD mice (Figure 2(d)). However, both insulin and leptin levels remained significantly higher in high-caloric treated mice during repair. *Ob/ob* mice did not produce the functional *ob* gene product [28, 29]. Basal TNF*α* levels were low in all animals, not dependent on obesity and not altered upon wounding (Figure 2(e)).

### 3.3. Prolonged Presence of Wound Macrophages and Neutrophils in HFD and *ob/ob* Mice

A controlled wound inflammation is pivotal to normal skin repair [30–32] and has been described to be augmented in *db/db* and *ob/ob* mice [13, 17]. Therefore, we determined the influx of macrophages (Figure 3) and neutrophils (Figure 4) into the healing tissue, as we hypothesized that increased numbers of leukocytes could be associated with the observed impaired healing conditions in HFD mice. To do so, we used the expression of distinct cellular markers restricted for macrophages (lysozyme M) or neutrophils (lipocalin), which represent constitutively expressed genes in these cell types and allow an overall quantification of cell numbers of the respective infiltrate at the wound site [13, 17]. In general, *ob/ob* mice exhibited the most severe dysregulation of macrophage and neutrophil influx: lysozyme M (Figure 3(a)) and lipocalin (Figure 4(a)) mRNA levels remained largely elevated upon injury. This was consistent with strong and long-lasting macrophage-specific F4/80 [33] (Figure 3(b), lower panel) or Ly6G (GR-1)-specific signals [34] (Figure 4(b), lower panel) in immunoblots from late wound tissue lysates. By contrast, the kinetics for lysozyme M and lipocalin mRNA expression were only moderately altered between CD and HFD mice (Figures 3(a) and 4(a)), but both the macrophage- or neutrophil-specific expression of the respective F4/80 (Figure 3(b)) and Ly6G (Figure 4(b)) proteins was markedly increased at the end of acute repair in HFD mice. This observation was also consistent with increased numbers of both cell types as assessed by immunohistochemical analysis of wound tissue (Figures 3(c) and 4(c)).

### 3.4. Expression of Proinflammatory Marker Proteins at the Wound Site

**Figure 5:**
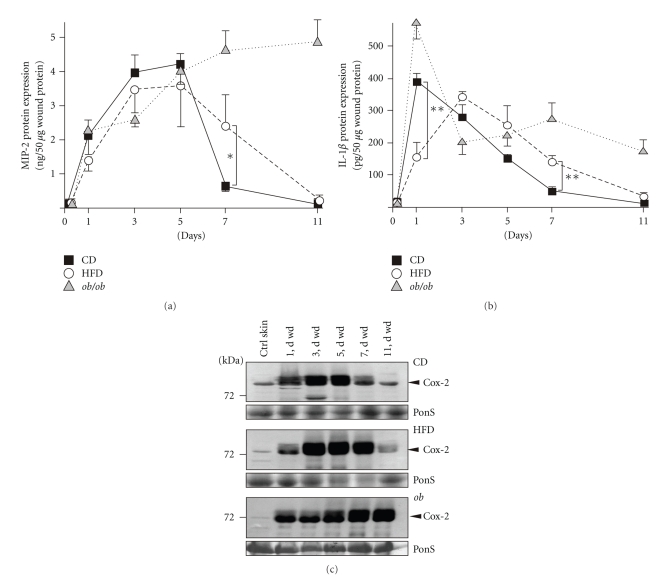
Wound inflammation. MIP-2 (a) and IL-1*β* (b) ELISA analyses from lysates of nonwounded back skin and lysates of wound tissue isolated from CD, HFD or *ob/ob* mice. Protein is expressed as ng (for MIP-2) or pg (for IL-1*β*) per 50 *μ*g skin or wound lysate. **P < .01; *P < .05 as indicated by the brackets. Each single experimental time point depicts the mean ± SD obtained from 8 wounds (*n* = 8) isolated from 4 individual animals (*n* = 4). (c) Immunoblots showing the presence of Cox-2 protein in nonwounded (ctrl) and wounded (1 to 11 d wd) skin in CD, HFD and *ob/ob* mice as indicated. Each time point depicts 8 wounds (*n* = 8) from 4 individual mice (*n* = 4). A Ponceau S (PonS) staining of the immunoblots is shown to control equal loading.

Next, we determined the expression pattern of the prototypic chemokine MIP-2 upon skin wounding, as chemokine release precedes the infiltration of leukocytes into the wound site [13, 17, 32, 35]. Again, *ob/ob *mice showed the most pronounced dysregulation of MIP-2 protein expression with highest levels in late wound tissue (day 5 and 7) (Figure 5(a)). Interestingly, the presence of MIP-2 protein started to differ not before the end of acute repair at day 5 after wounding between all experimental groups. In particular, the delayed healing in HFD mice was paralleled by still increased MIP-2 levels at day 7 of repair, when MIP-2 was nearly gone in CD mice (Figure 5(a)).

IL-1*β* is released from activated myeloid cells during acute inflammatory processes and represents an indicator molecule for inflammatory conditions [36–38]. IL-1*β* was induced upon wounding in CD, HFD and *ob/ob *mice, however, HFD mice revealed a delayed rise of this cytokine (Figure 5(b)). The acute wound response then exhibited comparable levels of IL-1*β* in CD and HFD mice. Again, IL-1*β* levels remained elevated particularly in 7-day late acute wounds of HFD and *ob/ob* mice. IL-1*β* then persisted in late chronic wounds of *ob/ob* mice, but declined to baseline in healing wounds of CD and HFD mice (day 11) (Figure 5(b)). 

Cox-2 is known to be differentially expressed in skin wounds [39]. Here, we observed the rapid induction of Cox-2 protein in all mice upon wounding (Figure 5(c)). In CD mice, Cox-2 expression started to decline after day 5 after wounding (Figure 5(c), upper panel). By contrast, Cox-2 protein remained increased also at day 7 of repair in HFD mice (Figure 5(c), middle panel), whereas *ob/ob* mice exhibited the most pronounced dysregulation even in late wound tissue (Figure 5(c), lower panel).

### 3.5. Wound Angiogenesis is Disturbed in HFD-Induced Obese and Diabetic Mice

**Figure 6:**
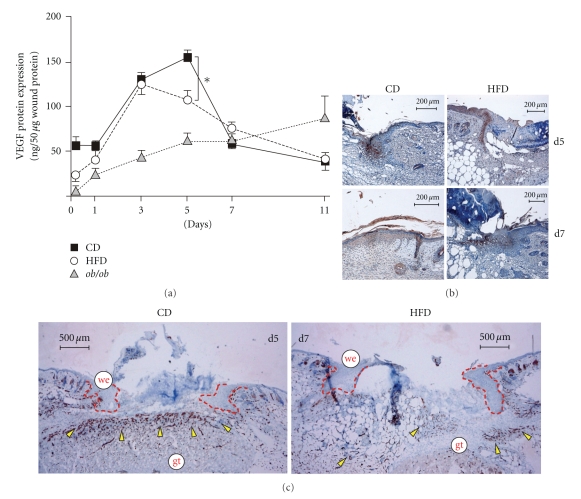
Angiogenic processes at the wound site. (a) VEGF ELISA analysis from lysates of nonwounded back skin and lysates of wound tissue isolated from CD, HFD or *ob/ob* mice. VEGF protein is expressed as ng per 50 *μ*g skin or wound lysate. *P < .05 as indicated by the bracket. Each single experimental time point depicts the mean ± SD obtained from 8 wounds (*n* = 8) isolated from 4 individual animals (*n* = 4). Sections from 5-day and 7-day wound tissue isolated from CD and HFD mice (as indicated) were stained for VEGF (b) or CD31 (c) Protein (*brown colour*). The epithelial margins are indicated by a *red line*. Scale bar equals 200 *μ*m in (b) or 500 *μ*m in (c). *gt*, granulation tissue; *we*, wound margin epithelia.

Angiogenic processes at the wound essentially contribute to normal skin repair [30, 31]. VEGF is pivotal to wound angiogenesis [40]. Thus, VEGF expression and wound angiogenesis have been shown to be severely impaired in *db/db* and *ob/ob* mice [16, 41, 42]. Here, we show that wound VEGF levels were only moderately different between CD and HFD mice at day 5 of repair (Figure 6(a)). Inline with published data [25, 43], it is important here that VEGF was expressed in wound margin keratinocytes (Figure 6(b)). The formation of well-developed, VEGF-expressing wound margin epithelia was delayed in HFD mice (Figure 6(b); refer also to Figure 2(b)). When wounds of CD mice were epithelialized at day 7 after wounding (Figure 6(b); refer to Figure 2(a)), wound margin epithelia of HFD mice still expressed VEGF protein (Figure 6(b), right panels). Using the endothelial marker CD31 to detect wound angiogenesis, we showed that wound VEGF signals appeared to be translated into a robust angiogenic response only in CD mice (Figure 6(c), left panel). By contrast, HFD mice exhibited a severely disturbed angiogenic response even at day 7 of repair (Figure 6(c), right panel).

### 3.6. Impaired Myofibroblast Differentiation and Wound Contraction in HFD-Induced Obese and Diabetic Mice

**Figure 7:**
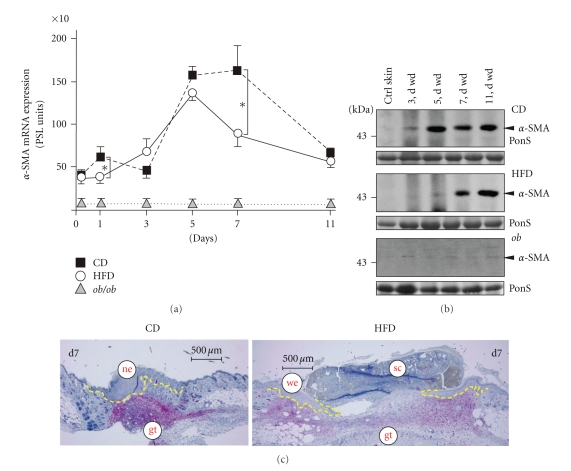
Myofibroblasts at the wound site. (a) Expression of *α*-SMA mRNA in nonwounded back skin and wound tissue isolated from CD-fed, HFD-fed or *ob/ob* mice. The time after injury is indicated. *P < .05 as indicated by the bracket. Each single experimental time point depicts the mean ± SD obtained from 12 wounds (*n* = 12) isolated from 4 individual animals (*n* = 4). (b) Immunoblots showing the presence of *α*-SMA protein in nonwounded (ctrl) and wounded (3 to 11 d wd) skin in CD, HFD and *ob/ob* mice as indicated. Each time point depicts 8 wounds (*n* = 8) from 4 individual mice (*n* = 4). A Ponceau S (PonS) staining of the immunoblots is shown to control equal loading. (c) Sections from 7-day wound tissue isolated from CD and HFD mice were stained for *α*-SMA protein as indicated (*red colour*). The epithelial margins are indicated by a *yellow line*. Scale bar = 500 *μ*m. gt, granulation tissue; ne, neoepithelium; sc, scab; we, wound margin epithelia.

Next, we investigated the appearance of myofibroblasts at the wound site, as we had observed a delay in wound contraction in HFD and *ob/ob* mice (Figure 2(a)). To this end, we assessed the *α*-SMA mRNA and protein expression (Figure 7) as a marker of the final differentiation of myofibroblasts [44]. The failure of fibroblasts to differentiate into myofibroblasts at the wound site in HFD mice could be shown by the delayed appearance of *α*-SMA mRNA (Figure 7(a), left panel) and protein (Figure 7(b)) in the animals. Here the *de novo* appearance of *α*-SMA protein was not visible earlier than day 7 of repair. Inline, immunohistochemistry showed myofibroblasts that were primarily located at the wound margins in HFD mice in the presence of a disturbed wound contraction (Figure 7(c), right panel). By contrast, epithelialized wounds of CD mice were characterized by a well-defined and myofibroblast-mediated contraction of wound tissue (Figure 7(c), left panel). We could not detect any *α*-SMA mRNA and protein expression in wounds of *ob/ob* mice throughout the complete period of healing (Figures 7(a) and 7(b)).

### 3.7. Modified Activation of Inflammatory Signaling Pathways in Obese and Diabetic Mice

**Figure 8:**
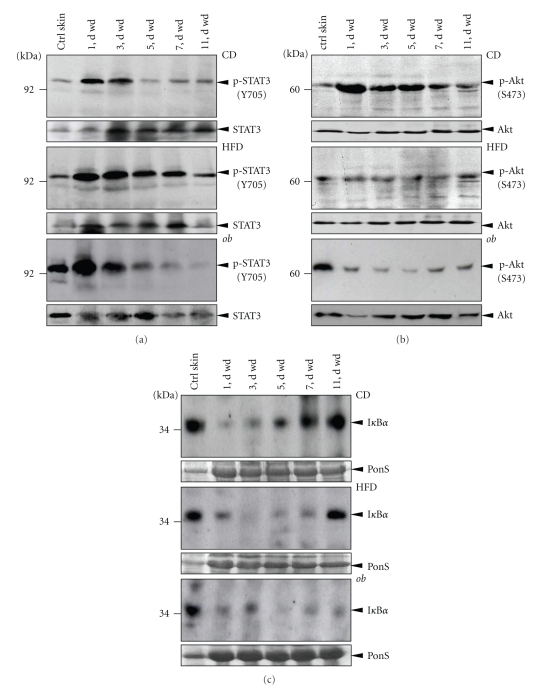
Activation of signaling pathways in wound tissue. Immunoblots showing phosphorylated STAT3 (a) and Akt (b) or the presence of I*κ*B*α* (c) in nonwounded (ctrl skin) and wounded (1 to 11 d wd) skin in CD, HFD and *ob/ob* mice as indicated. Each time point depicts 8 wounds (*n* = 8) from 4 individual mice (*n* = 4). Total STAT3 and Akt or a Ponceau S staining (as indicated) were shown to control equal loading.

Finally, we investigated central prototypical signaling pathways in normal and impaired wound tissue. The activation of STAT-3 [45], Akt [42] and nuclear factor *κ*B (NF*κ*B) [46, 47] is implicated in skin wounds. Here we observed a STAT3 activation kinetic that appeared independent from the obese and diabetic phenotype: STAT3 phosphorylation (Y705) was rapidly induced upon injury and the activation started to decline from day 3 after wounding (Figure 8(a)). By contrast, a prominent activation of Akt (S473 phosphorylation) was limited to the acute inflammatory phase of repair in lean CD mice, whereas an Akt activation was absent in wound tissue from obese and diabetic HFD and *ob/ob* mice throughout the complete period of healing (Figure 8(b)). Prior to activation of the classical proinflammatory NF*κ*B signalling pathway, the I*κ*B*α* protein has to be introduced to proteasomal degradation to release NF*κ*B proteins for active signalling [47]. Here the disappearance of I*κ*B*α* protein paralleled the acute wound inflammatory phase in lean CD mice. The I*κ*B*α* protein became visible again in 5-day wound tissue in the animals, that time point when the resolution of wound inflammation normally occurs (Figure 8(c), upper panel). However, a prominent I*κ*B*α* signal was still missing in HFD mice at that time point of repair (Figure 8(c), middle panel). Moreover, *ob/ob* mice showed an absence of I*κ*B*α* over time and thus an activation of the NF*κ*B pathway even in late wounds (Figure 8(c), lower panel).

## 4. Discussion

Diabetic ulcerations remain a still unresolved clinical condition with a high mortality [2, 3], that will become more significant with the expected rise in the prevalence of type 2 diabetes in the next two decades [48]. The failure of pharmacologic treatment options, with platelet-derived growth factor-BB as the only exception [49], further adds to this disappointing clinical situation. Chronic wounds represent diverse cutaneous lesions that do not undergo the coordinated progress of normal tissue repair [50]. Despite the basic necessity of a controlled wound inflammation, granulation tissue formation and reepithelialization [30, 31], diabetes-impaired wounds appear to have lost the ideal synchrony of events that lead to rapid healing [50]. Although important intrinsic (neuropathy, vascular problems) or extrinsic (wound infection, callus formation, pressure to the site) factors of human diabetic ulceration [50] can only be stimulated to some extent in animals, there is no alternative to the use of wound healing models in type 2 diabetic rodents. Therefore, much of important knowledge with respect to underlying mechanisms of the diabetic ulceration has been achieved by using diabetic mice. Early basic papers in the field of wound healing established the use of the diabetic *ob/ob* [9] and *db/db* [11] mouse as models for impaired wound healing. However, syndromes in both the *db/db* and *ob/ob* mouse were not limited to insulin-resistance only. The underlying genetic defect in leptin signaling in the animals [29, 51] additionally causes hyperphagia, hyperinsulinemia, hyperglycemia and a marked obesity [8]. These phenotypic changes are mediated by the loss of leptin action in the hypothalamus, where the cytokine normally regulates neuronal and endocrine circuits [52]. For these reasons, the respective genetic defects in *db/db* and *ob/ob* mice mainly combine a pronounced obesity with an insulin-resistance, actually generating a model of metabolic syndrome [53]. 

This is all the more interesting, as obesity-associated insulin-resistance is currently regarded as a consequence of persisting low-grade inflammatory conditions that originate in a pathologically altered communication of adipose tissue leading to macrophage activation [20–22]. Indeed, the pathophysiologic interconnection between the marked adipose tissue and macrophage activation exhibits functional consequences for skin repair in *ob/ob* mice: depletion of wound macrophages by different techniques (antibodies, clodronate liposomes) markedly improved tissue regeneration in *ob/ob* mice [18, 23, 54]. Hence, these studies strongly suggest that the marked signs of obesity, that were inextricably connected to the type 2 diabetes in the genetically diabetic and obese *ob/ob* mouse [8, 29], pivotally contribute to the observed wound disturbances in the animals.

Therefore, it was reasonable here to investigate cutaneous tissue repair in a diet-induced mouse model of obesity and insulin-resistance. Such a study would allow to determine potential differences, advantages or short-cuts in the use of genetically diabetic and obese mice in wound healing experiments. Our 6-month HFD treatment of mice resulted in elevated blood glucose and insulin levels in the animals. This is in accordance to established findings showing that a 6-month *ad libitum* exposure to a high-fat, simple carbohydrate diet produced obesity, hyperglycemia and hyperinsulinemia in C57Bl/6J mice [55]. However, it becomes obvious that the loss of functional leptin in *ob/ob* mice [29] resulted in more severe hyperglycaemic and insulinaemic states [8, 53]. Increased serum leptin levels in HFD-fed mice reflect the growing adipose tissue in obese animals [56]. Thus, the determination of endocrine and physiologic parameters strongly showed that HFD-feeding had resulted in the development of an obese and diabetic phenotype in C57Bl/6J mice.

Not unexpected, our wounding of HFD mice resulted in a general delay in wound closure. Three main findings with respect to tissue repair in HFD mice have to be accentuated here. First, the main disturbances affected the acute wound inflammatory response, which appeared to be prolonged. Second, the formation of normal wound margin epithelia was disturbed, and third, we observed a marked impairment in myofibroblast differentiation and thus wound contraction. Here, the loss of myofibroblast differentiation in wound tissue appeared to be the most prominent difference between the genetic and the induced mouse model of diabetes. The absence of myofibroblasts in wounds of *ob/ob* mice might reflect a direct functional consequence of a strongly impaired transforming growth factor (TGF)-*β* signalling in disturbed wound tissue in the animals. TGF-*β*1 represents the essential signal to induce the formation of contractile bundles in normal wound fibroblasts [44]. Therefore, it is tempting to argue that an augmented wound inflammation in diabetic mice might drive alterations in the responsiveness of wound fibroblasts to external signals, as fibroblasts isolated from chronic wounds revealed a disturbed signaling from the TGF-*β*II receptor [57]. Inline with this argumentation, chronic ulcers in humans were characterized by the suppression of TGF-*β* receptors I-III and the subsequent absence of Smad2 phosphorylation [58]. In addition, TNF-*α*, which exhibits a markedly prolonged and elevated presence in disturbed wounds of *ob/ob* mice [17], has been shown to potently suppress TGF-*β*1-induced myofibroblast phenotypic genes in fibroblasts [59]. Thus, the strongly exacerbated wound inflammation in *ob/ob* mice (in the study, [17]) appears to contribute to the observed extensive loss of myofibroblast differentiation and wound contraction as a consequence.

In addition, wounding of mice resulted in a noticeable decrease in circulating insulin and leptin levels, which reached basal levels again at the end of repair. Although published data dealing with systemic effect of trauma are limited, our finding appeared consistent with acutely reduced insulin levels in rats after surgical trauma [60]. Moreover, injury-mediated decreasing levels of leptin clearly indicate that circulating levels of this cytokine were not simply a result of adipose tissue mass [56], but also susceptible to an acute systemic response such as trauma (wounding).

The HFD-induced impairments in wound inflammation, reepithelialization, angiogenesis and contraction have to be considered in relation to published data from wounded *ob/ob* mice. In conclusive analogy to conditions in HFD-treated mice, wounded *ob/ob* mice also exhibited reduced wound margin epithelia [10], increased levels of MIP-2, IL-1*β* [17] and Cox-2 [61] protein, a prolonged presence of neutrophils and macrophages at the wound site [17, 18], moderately altered wound VEGF levels [41] or impaired signaling pathways [54]. Due to the overall consistence of wound conditions in both HFD and *ob/ob* mice, it is actually allowed here to state that the deleterious effects of obesity and diabetes caused a more pronounced impairment and also delayed healing in *ob/ob* mice. Our findings from *ob/ob* mice show that wound inflammation as well as reepithelialization were severely impaired even throughout late repair. Thus, the underlying leptin deficiency in *ob/ob* mice further amplified those impairments in skin repair that could also be observed in the diet-induced obese and diabetic mouse. Therefore, our data support the general use of the *ob/ob* mouse model to investigate basic mechanisms of diabetes-disturbed skin repair. As long as these differences were considered with respect to a careful and thorough interpretation of data, the *ob/ob* mouse holds the strong advantage to show an amplification of particularly those injury-induced inflammatory and also tissue responses that were less strongly developed in a diet-induced mouse model of obesity and diabetes.

## Abbreviations

*α*-SMA: *α*-smooth muscle actin
CD: Chow diet
Cox-2: Cyclooxygenase-2
HFD: High-fat diet
I*κ*B*α*: Inhibitor of nuclear factor *κ*B*α*
MIP: Macrophage inflammatory protein
OGT: Oral glucose tolerance test
PECAM-1: Platelet endothelial cell adhesion molecule
STAT: Signal transducer and activator of transcription
VEGF: Vascular endothelial growth factor.
